# Global burden of colorectal cancer mortality by age and sex, 1990–2050: a population-based analysis

**DOI:** 10.7189/jogh.16.04383

**Published:** 2026-11-06

**Authors:** Tingfan Luo, Weihong Chen, Yaru Cao, Pengting Li, Jin Feng

**Affiliations:** 1 Department of Radiation Oncology, Sun Yat-sen University Cancer Center Gansu Hospital, Lanzhou, China; 2 Department of Emergency Medicine, The Second Clinical Medical College of Lanzhou University, Lanzhou, China; 3 Department of Radiation Oncology, The Second Clinical Medical College of Lanzhou University, Lanzhou, China; 4 Department of Emergency Medicine, The First People’s Hospital of Longnan, Longnan, China; 5 NHC Key Laboratory of Environment and Endemic Diseases, School of Public Health, Health Science Center, Xi'an Jiaotong University, Xi’an, China

**Keywords:** colorectal cancer, Global Burden of Disease 2021, age-specific mortality, risk factors, projection

## Abstract

**Background:**

Colorectal cancer (CRC) represents a major global public health challenge, yet age- and sex-specific patterns in mortality burden and risk factor contributions remain incompletely characterised. We aimed to analyse global age- and sex-specific mortality patterns of CRC from 1990 to 2021, project trends to 2050, and assess the mortality burden attributable to risk factors.

**Methods:**

We extracted CRC mortality data from 204 countries and territories between 1990 and 2021 from Global Burden of Disease 2021. We stratified the population into very early-onset CRC (VEOCRC) (aged 15–34 years), early-onset CRC (EOCRC) (aged 35–49 years), and late-onset CRC (LOCRC) (aged 50–95 years). Age-specific mortality rates (MRs) characterised mortality burden, and estimated annual percentage changes quantified temporal trends. We applied population attributable fractions (PAFs) to estimate the mortality burden attributable to risk factors and used Bayesian age–period–cohort models to project global mortality trends from 2022 to 2050.

**Results:**

From 1990 to 2021, LOCRC accounted for the greatest global mortality burden, followed by EOCRC and VEOCRC. The VEOCRC and EOCRC MRs were highest in East Asia, while LOCRC rates were highest among females in Australasia and males in Western Europe. All three subtypes had the highest MRs in the high-middle sociodemographic index region. Males consistently had higher MRs than females across all three subtypes. Generally, MRs declined from 1990 to 2021 across all three CRC subtypes, although some low- and middle-income regions, particularly East Asia, Latin America, and Africa, showed increasing trends. The PAFs of risk factors increased with age, particularly for behavioural, metabolic, and dietary factors. Projections suggested declining global CRC age-standardised MRs by 2050, while LOCRC would remain the major contributor, with rates of 57.47 per 100,000 in males and 31.40 in females.

**Conclusions:**

We found substantial age, sex, and regional disparities in global CRC mortality. Despite projected declines by 2050, LOCRC will remain the dominant contributor, while younger-onset CRC warrants greater attention.

Colorectal cancer (CRC) is a common malignancy of the digestive system that primarily involves the colon and rectum [1]. It is the third most diagnosed cancer and the second leading cause of cancer-related mortality worldwide [2]. In 2020, approximately 1.9 million new CRC cases and over 930,000 deaths were estimated worldwide [3]. Although advances in systemic and personalised therapies have substantially improved patients’ survival, the overall prognosis remains poor [4]. A substantial burden is imposed by CRC on patients and healthcare systems. However, previous studies have largely focused on overall CRC burden, with limited evidence on age- and sex-specific mortality patterns and their future trajectories.

Over the past several decades, CRC mortality burden has remained stable or declined in high-income countries, largely due to advances in treatment strategies and shifts in the distribution of key risk factors [5]. Previous studies show that CRC is strongly associated with age, with substantial differences in disease burden and epidemiological characteristics between sexes [6]. Early-onset CRC (EOCRC) is commonly defined as CRC diagnosed before age 50, whereas CRC diagnosed at or after age 50 is referred to as late-onset CRC (LOCRC) [7]. Based on previous definitions, EOCRC can be further stratified, with cases diagnosed before age 35 classified as very early-onset CRC (VEOCRC) [8]. Based on Global Burden of Disease (GBD) 2019 and 2021, previous studies have demonstrated substantial heterogeneity in the burden of EOCRC across the globe, sociodemographic index (SDI) regions, age groups, and sexes, and further projected future trends in disease burden [9,10]. Dietary risk factors and high body mass index (BMI) have been the primary focus of risk factor assessments for EOCRC in both GBD 2019 and 2021 studies. These studies consistently showed that higher intakes of whole grains and dietary fibre, and lower consumption of red and processed meats were associated with a lower risk of EOCRC [11,12]. By integrating data from the SEER 17 and the GBD 2021, Jin *et al.* found significant differences in clinicopathological characteristics, prognosis, and epidemiological patterns between patients with CRC aged 15–29 years and those aged 30–49 years, with patients aged 15–29 years exhibiting more aggressive pathological features, poorer prognosis, and a higher global incidence of CRC [13]. Previous GBD studies show that, between 1990 and 2021, LOCRC continued to account for most CRC cases worldwide, and the burden of high BMI-attributable LOCRC consistently exceeded that of EOCRC. However, the burden of EOCRC attributable to high BMI increased more rapidly over time [14,15].

Existing studies have primarily evaluated the overall burden of CRC using age-standardised measures, whereas age-specific mortality patterns across the life course remain insufficiently characterised. A systematic, comprehensive assessment of mortality burden across VEOCRC, EOCRC, and LOCRC is lacking. To further characterise age-specific patterns within EOCRC, we defined CRC diagnosed at ages 15–34 years as VEOCRC and at ages 35–49 years as EOCRC. To address this knowledge gap, we aimed to systematically assess the age-specific mortality burden of three CRC subtypes using GBD 2021 data, providing a more comprehensive understanding of age-related heterogeneity in the global CRC mortality burden and offering evidence to inform age-specific prevention and control strategies.

We aimed to systematically evaluate CRC mortality rates (MRs) (per 100,000 persons) and temporal trends by sex, subtype, and geographic region. We further explored associations between regional CRC MRs and socioeconomic development levels. In addition, we systematically analysed CRC mortality risk factors by subtype and sex and projected mortality burden trends from 2022 to 2050.

## METHODS

We adhered to the Journal of Global Health’s GRABDROP guidelines (Table S1 in the **Online Supplementary Document**) [16].

### Data source

Covering 204 countries and territories, GBD 2021 provides epidemiological estimates for 369 diseases and injuries, quantifies health losses attributable to 88 risk factors, and reports key measures across 23 age groups from birth to ≥95 years for males, females, and both sexes [17]. The GBD framework integrates multiple sources, including vital registration systems, cancer registries, verbal autopsy studies, and other available data, and applies standardised modelling approaches such as the Cause of Death Ensemble model to generate comparable mortality estimates across locations and time periods. The SDI is a composite measure reflecting regional sociodemographic development, with values ranging from 0 to 1, and SDI values in the GBD 2021 were multiplied by 100 to generate a standardised scale from 0 to 100 [18]. Human Development Index (HDI) data for all countries from 1990 to 2021 were obtained from the Human Development Reports Data Center [19]. Diseases were coded and classified according to the ICD framework in the GBD, with cancers being categorised into 34 types.

We obtained data on CRC (ICD-10 codes C18–21.9) mortality, along with their corresponding 95% uncertainty intervals (UIs), spanning 1990–2021 at the global level, across five SDI regions, and for 204 countries and territories. In addition, the extracted data included age groups from 15 to ≥95 years for males and females. The data were accessible using the Global Health Data Exchange query tool [20]. We used publicly available aggregated data from the GBD 2021 study and did not use any individual-level data.

### Risk factors

We extracted all CRC-related risk factors from the GBD 2021 database for 1990-2021 globally and across five SDI regions. We selected deaths as the outcome measure to quantify each risk factor's contribution to CRC mortality burden. We extracted data for males and females across age groups from 15 to ≥95 years. The GBD uses a comparative risk assessment framework to estimate the attributable burden of each risk factor by estimating relative risks, defining the theoretical minimum risk exposure level, and calculating population attributable fractions (PAFs) [21,22]. The combined PAF for each predefined age group was calculated as follows:



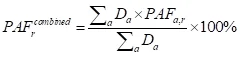



Where *PAF_r_^combined^* represents the combined PAF for risk factor *r* across the target age range; *D_a_* represents the total number of CRC deaths in age group *a*; *PAF_a,r_* represents the age-specific PAF of risk factor *r* for CRC deaths in age group *a*; *a* denotes the age group within the predefined age range (15–34, 35–49, or 50–95 years); and *r* represents the evaluated risk factor.

### Bayesian age-period-cohort analysis

The Bayesian age–period–cohort (BAPC) model integrates age, period, and cohort effects to project future trends in disease burden and distinguish their respective contributions to age-specific rates [23–25]. If the observed number of deaths follows a Poisson distribution:

*Y*_ap_ ~ *Poisson*(*N_ap_r_ap_*)

where *Y*_ap_ represents the observed number of deaths for age group *a* in period *p*; *N_ap_* represents the corresponding population at risk; and *r_ap_* represents the age-specific rate for age group *a* in period *p*.



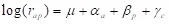



where *α_a_*, *β_p_*, and *γ_c_* denote age, period, and cohort effects, respectively. We assigned a second-order random walk prior to these effects and estimated the model using INLA.



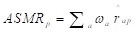



*ASMR_p_* represents the age-standardised MR (ASMR) in period *p*, *ω_a_* denotes the proportion of the standard population in age group *a*, and *r̂_ap_* indicates the predicted age-specific MR for age group *a* in period *p*. We derived the 95% UIs from the 2.5th and 97.5th percentiles of the posterior distribution. Due to prediction uncertainty, some lower bounds of UIs for very low predicted rates may fall below 0, reflecting estimation uncertainty rather than biologically meaningful negative MRs.

### Statistical analysis

We categorised countries into low-, middle-, and high-HDI groups by HDI tertiles. Age-specific death rates were calculated for females and males by aggregating deaths and populations across the age groups within the predefined age ranges, as follows:



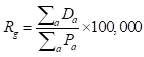



where *R_g_* represents the age-specific rate for the predefined age range *g*; *D_a_* denotes the number of deaths in age group *a*; *P_a_* denotes the corresponding population in age group *a*; and *a* represents each age group within the *g*. The estimated annual percentage change (EAPC) was used to assess the temporal trends in age-specific death rates across the study period and was calculated as follows:



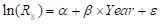





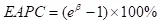


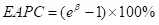



where *α* is the intercept; *β* is the regression coefficient for calendar year; *Year* denotes the calendar year; and *ε* is the random error term. We obtained the 95% confidence interval (CI) of the EAPC by applying the same exponential transformation to the lower and upper limits of the 95% CI of *β*. We considered an increasing trend statistically significant when the lower limit of the 95% CI for the EAPC was greater than 0, whereas a decreasing trend was statistically significant when the upper limit was less than 0. Otherwise, we considered the trend statistically non-significant.

We used *R*, version 4.3.2 (R Core Team, Vienna, Austria) for all analyses. We used the BAPC and INLA packages to perform the BAPC analysis.

## RESULTS

### Global and regional mortality burden of CRC subtypes

Compared with 1990 and 2005, MRs for VEOCRC, EOCRC, and LOCRC decreased in both males and females (**Table 1**). Across all periods, MRs were highest for LOCRC, followed by EOCRC and VEOCRC, and males consistently had higher MRs than females across all three CRC onset categories. Across the globe and the 21 GBD regions at the three time points (*i.e.* 1990, 2005, and 2021), MRs for the three CRC subtypes showed substantial geographic variation. East Asia consistently had the highest VEOCRC and EOCRC MRs in both females and males. In contrast, the lowest VEOCRC MRs among females were observed in Oceania in 1990 (MR = 1.18; 95% UI = 0.71, 1.95) and 2005 (MR = 1.18; 95% UI = 0.74, 1.88), whereas the lowest rate among males was observed in South Asia in 1990 (MR = 1.15: 95% UI = 0.77, 1.84). For EOCRC, females in Oceania (MR = 5.04; 95% UI = 3.15, 8.05) and males in South Asia (MR = 6.45; 95% UI = 4.40, 9.79) had the lowest MRs in 1990. For LOCRC, Central Sub-Saharan Africa consistently had the lowest MRs for both females (MR = 208.40; 95% UI = 119.03, 349.06) and males (MR = 253.20; 95% UI = 152.90, 434.35) in 1990. Conversely, females in Australasia (MR = 2295.35; 95% UI = 1774.42, 2767.38) and males in Western Europe (MR = 3038.82; 95% UI = 2670.01, 3333.27) had the highest rates.

**Table 1:** MRs of VEOCRC, EOCRC, and LOCRC across regions and years*

	15–34 years		35–49 years		50–95 years	
	**Female**	**Male**	**Female**	**Male**	**Female**	**Male**
**Andean Latin America**						
2005	2.25 (1.74, 2.91)	2.60 (2.03, 3.32)	10.87 (8.78, 13.33)	10.79 (8.60, 13.36)	1,000.19 (785.01, 1261.79)	859.54 (677.32, 1061.02)
2021	2.51 (1.96, 3.24)	2.92 (2.32, 3.67)	12.90 (10.54, 16.05)	13.46 (10.62, 16.84)	1,023.98 (801.70, 1340.17)	970.48 (790.11, 1183.74)
1990	1.77 (1.38, 2.32)	2.16 (1.65, 2.92)	9.03 (7.15, 11.72)	8.36 (6.48, 11.13)	800.70 (612.41, 1098.21)	664.83 (505.41, 879.57)
**Australasia**						
2005	2.00 (1.61, 2.48)	2.49 (2.06, 3.07)	15.19 (12.93, 18.13)	17.27 (14.64, 20.28)	1,885.59 (1,393.00, 2340.58)	2,355.74 (1,932.53, 2766.32)
2021	1.69 (1.37, 2.06)	1.93 (1.54, 2.40)	14.51 (12.18, 17.11)	15.16 (12.61, 18.14)	1,733.81 (1,231.02, 2156.64)	2,040.56 (1,634.71, 2456.63)
1990	2.56 (2.09, 3.22)	2.80 (2.30, 3.37)	22.20 (18.74, 26.39)	24.22 (20.67, 28.50)	2295.35 (1774.42, 2767.38)	2849.42 (2371.31, 3326.15)
**Caribbean**						
2005	2.25 (1.77, 2.96)	2.78 (2.14, 3.62)	12.51 (10.66, 14.79)	13.05 (11.15, 15.55)	1,297.51 (1,076.94, 1509.22)	1,149.87 (975.06, 1328.63)
2021	3.31 (2.41, 4.49)	3.85 (2.91, 5.23)	16.86 (13.78, 21.13)	18.55 (15.33, 22.91)	1,510.98 (1,259.13, 1791.85)	1,464.99 (1,233.32, 1746.92)
1990	2.99 (2.43, 3.76)	3.41 (2.77, 4.29)	15.87 (13.38, 18.97)	15.57 (13.08, 18.53)	1229.57 (1034.07, 1430.70)	1124.90 (943.55, 1322.80)
**Central Asia**						
2005	2.22 (1.83, 2.75)	2.94 (2.43, 3.56)	10.78 (9.25, 12.63)	12.87 (10.93, 15.02)	405.59 (342.07, 475.80)	535.49 (453.90, 630.85)
2021	1.62 (1.29, 2.07)	2.04 (1.59, 2.60)	9.01 (7.38, 11.16)	10.90 (9.05, 13.29)	470.06 (379.47, 572.91)	679.36 (554.52, 825.11)
1990	3.31 (2.83, 3.85)	4.56 (3.96, 5.22)	16.39 (14.37, 18.65)	18.76 (16.55, 21.12)	561.14 (475.37, 650.36)	748.88 (644.73, 865.28)
**Central Europe**						
2005	2.21 (1.93, 2.55)	2.88 (2.52, 3.30)	18.59 (16.72, 20.76)	26.67 (24.30, 29.40)	1,821.56 (1,528.31, 2056.29)	2,761.07 (2,437.78, 3053.48)
2021	1.87 (1.67, 2.10)	2.25 (2.00, 2.53)	14.83 (13.34, 16.59)	20.26 (18.39, 22.40)	1,551.74 (1,327.31, 1737.91)	2,783.86 (2,483.05, 3043.96)
1990	2.61 (2.33, 2.92)	3.14 (2.79, 3.55)	20.23 (18.59, 22.08)	26.70 (24.54, 28.99)	1639.42 (1399.83, 1852.33)	2239.47 (1994.88, 2488.52)
**Central Latin America**						
2005	2.06 (1.75, 2.41)	2.75 (2.36, 3.22)	9.56 (8.44, 10.79)	10.83 (9.45, 12.52)	700.28 (576.78, 816.23)	626.95 (530.93, 726.22)
2021	2.47 (2.12, 2.86)	3.20 (2.73, 3.75)	12.81 (11.32, 14.50)	15.55 (13.61, 17.88)	764.59 (646.79, 875.59)	794.05 (686.59, 908.61)
1990	1.94 (1.65, 2.33)	2.29 (1.94, 2.67)	9.04 (8.16, 10.15)	8.60 (7.57, 9.64)	705.43 (577.07, 832.46)	574.34 (489.19, 650.79)
**Central Sub-Saharan Africa**						
2005	2.82 (1.43, 5.20)	1.65 (0.89, 2.92)	10.04 (5.07, 17.93)	6.52 (3.44, 11.25)	212.62 (103.03, 376.51)	259.26 (134.01, 451.81)
2021	4.88 (2.38, 9.23)	1.88 (0.97, 3.65)	19.76 (9.55, 37.33)	10.08 (5.22, 18.69)	278.92 (127.06, 540.17)	394.91 (199.78, 742.59)
1990	3.98 (2.25, 6.82)	2.05 (1.22, 3.66)	11.27 (6.17, 19.63)	6.89 (4.23, 11.72)	208.40 (119.03, 349.06)	253.20 (152.90, 434.35)
**East Asia**						
2005	3.88 (2.83, 5.39)	7.52 (5.47, 10.26)	14.82 (11.28, 20.30)	31.17 (23.53, 41.11)	870.72 (615.04, 1186.29)	1,361.64 (1,005.47, 1779.95)
2021	2.04 (1.50, 2.96)	3.84 (2.84, 5.22)	9.27 (7.10, 13.29)	23.10 (18.09, 29.99)	801.18 (555.55, 1147.28)	1333.58 (1011.74, 1742.49)
1990	7.43 (4.40, 10.49)	8.90 (5.70, 13.26)	23.41 (14.86, 32.32)	31.47 (22.38, 43.60)	951.32 (645.80, 1378.03)	1324.58 (929.55, 1887.81)
**Eastern Europe**						
2005	2.79 (2.29, 3.42)	2.95 (2.45, 3.53)	19.84 (16.79, 23.71)	21.97 (18.65, 25.73)	1,109.77 (919.94, 1310.41)	1,805.21 (1,540.89, 2086.39)
2021	1.79 (1.50, 2.20)	2.11 (1.74, 2.63)	15.37 (13.01, 18.68)	19.89 (16.87, 23.56)	1,237.58 (1,009.21, 1477.05)	1,826.70 (1,544.83, 2153.46)
1990	2.66 (2.21, 3.19)	3.06 (2.57, 3.64)	20.50 (17.62, 24.38)	21.76 (18.69, 25.38)	1016.84 (855.45, 1196.22)	1474.88 (1277.35, 1710.42)
**Eastern sub-Saharan Africa**						
2005	3.68 (2.42, 5.39)	4.07 (2.54, 6.03)	12.66 (8.58, 18.54)	12.87 (8.24, 18.89)	447.10 (281.73, 678.60)	741.08 (446.08, 1105.27)
2021	4.34 (2.69, 6.81)	4.90 (3.00, 7.68)	17.99 (11.48, 26.21)	18.87 (11.42, 28.48)	552.86 (340.55, 849.17)	1,348.16 (832.60, 2093.76)
1990	5.41 (3.54, 8.20)	5.82 (3.81, 9.39)	15.37 (10.08, 22.71)	16.49 (11.00, 25.48)	398.83 (249.44, 620.38)	608.18 (394.40, 984.44)
**Global**						
2005	2.61 (2.13, 3.22)	3.59 (2.97, 4.31)	12.96 (11.24, 15.11)	18.47 (16.09, 21.07)	1,324.33 (1,034.19, 1533.03)	1,593.80 (1,361.88, 1767.51)
2021	2.52 (1.94, 3.21)	2.95 (2.35, 3.70)	12.50 (10.23, 15.17)	16.74 (14.24, 19.71)	1,168.87 (889.39, 1374.45)	1,512.34 (1,287.42, 1705.59)
1990	3.63 (2.64, 4.47)	4.15 (3.20, 5.23)	16.16 (13.16, 18.92)	19.07 (16.19, 22.42)	1445.85 (1160.54, 1653.35)	1664.47 (1442.39, 1859.83)
**High-income Asia Pacific**						
2005	2.01 (1.67, 2.44)	2.35 (1.97, 2.81)	14.26 (12.37, 16.62)	18.22 (16.05, 21.03)	1,878.49 (1,282.58, 2388.42)	2,703.59 (2,194.35, 3206.02)
2021	1.68 (1.38, 2.03)	**1**.97 (1.56, 2.46)	11.48 (9.81, 13.43)	13.94 (11.92, 16.59)	2,027.98 (1,317.15, 2618.16)	2,734.57 (2,161.16, 3290.78)
1990	2.95 (2.31, 3.61)	3.55 (2.87, 4.30)	20.18 (17.51, 23.34)	25.97 (22.34, 30.19)	1564.00 (1123.48, 1931.14)	2481.97 (2037.70, 2920.11)
**High-income North America**						
2005	1.97 (1.53, 2.46)	2.43 (1.93, 3.08)	14.82 (12.43, 17.58)	18.92 (15.93, 22.46)	1,595.66 (1,131.98, 2045.80)	2,043.15 (1,563.00, 2489.95)
2021	2.10 (1.67, 2.62)	2.82 (2.18, 3.62)	16.97 (14.41, 19.84)	22.37 (18.97, 26.51)	1,261.53 (913.53, 1565.14)	1,506.81 (1,181.01, 1824.08)
1990	2.00 (1.63, 2.55)	2.50 (2.04, 3.02)	15.78 (13.52, 18.73)	19.98 (17.09, 23.16)	1916.89 (1409.55, 2373.98)	2531.45 (2029.08, 3006.80)
**North Africa and Middle East**						
2005	2.30 (1.69, 3.19)	2.40 (1.71, 3.30)	11.13 (8.60, 14.78)	12.00 (8.90, 15.51)	710.14 (516.59, 989.60)	902.90 (651.42, 1196.12)
2021	2.69 (1.88, 3.67)	2.84 (2.07, 3.83)	11.68 (8.92, 14.88)	10.97 (8.63, 13.63)	832.63 (645.60, 1053.06)	1,183.47 (892.25, 1525.19)
1990	2.16 (1.47, 3.17)	2.53 (1.62, 4.08)	10.99 (7.75, 15.60)	12.22 (8.03, 18.71)	649.41 (430.39, 951.74)	733.25 (473.83, 1147.23)
**Oceania**						
2005	1.18 (0.74, 1.88)	3.51 (2.05, 5.86)	5.53 (3.64, 8.44)	10.21 (6.45, 15.29)	449.47 (289.12, 684.93)	750.38 (474.75, 1136.62)
2021	1.71 (0.98, 2.78)	4.57 (2.34, 8.31)	6.51 (4.00, 10.00)	11.43 (6.54, 19.37)	487.77 (315.65, 724.84)	872.03 (510.95, 1404.03)
1990	1.18 (0.71, 1.95)	3.61 (2.09, 6.32)	5.04 (3.15, 8.05)	10.81 (6.67, 17.63)	463.39 (299.09, 724.42)	819.25 (536.84, 1297.47)
**South Asia**						
2005	1.76 (1.16, 2.71)	1.35 (0.89, 1.96)	8.41 (5.41, 12.59)	6.77 (4.74, 9.38)	314.28 (207.54, 488.09)	329.21 (213.53, 470.43)
2021	2.21 (1.42, 3.29)	2.06 (1.29, 3.24)	9.85 (6.50, 14.50)	8.86 (5.89, 13.22)	364.80 (241.94, 547.56)	458.27 (303.62, 694.81)
1990	1.88 (1.18, 2.86)	1.15 (0.77, 1.84)	9.15 (5.74, 14.09)	6.45 (4.40, 9.79)	270.80 (171.76, 407.60)	273.78 (178.57, 426.06)
**Southeast Asia**						
2005	3.20 (2.18, 4.69)	4.62 (3.22, 6.40)	13.93 (10.17, 19.60)	21.18 (15.84, 27.57)	592.27 (428.86, 815.74)	850.06 (630.04, 1101.99)
2021	4.27 (2.82, 6.12)	4.89 (3.36, 7.27)	19.75 (13.48, 27.58)	27.94 (19.65, 39.64)	607.49 (430.61, 840.07)	990.46 (716.86, 1348.67)
1990	2.45 (1.66, 3.61)	3.62 (2.44, 5.68)	13.32 (9.49, 18.94)	16.34 (11.42, 24.53)	497.27 (343.94, 727.55)	684.66 (465.76, 1028.77)
**Southern Latin America**						
2005	2.67 (2.18, 3.25)	3.28 (2.74, 4.02)	16.07 (13.67, 18.98)	20.34 (17.45, 23.82)	2,032.28 (1,592.51, 2459.09)	2,586.13 (2,172.90, 3038.09)
2021	3.01 (2.48, 3.66)	3.52 (2.89, 4.32)	18.23 (15.73, 21.32)	22.33 (18.99, 26.19)	1,661.83 (1,322.68, 1956.53)	2,213.21 (1,877.33, 2578.71)
1990	2.68 (2.24, 3.30)	2.90 (2.43, 3.45)	16.87 (14.54, 19.98)	21.65 (18.63, 25.10)	1920.89 (1526.01, 2293.20)	2391.95 (2047.15, 2780.29)
**Southern Sub-Saharan Africa**						
2005	2.20 (1.46, 3.17)	2.48 (1.72, 3.64)	7.92 (5.65, 11.28)	10.86 (7.92, 14.76)	540.51 (331.19, 780.47)	774.10 (558.20, 1047.11)
2021	1.86 (1.12, 2.87)	2.10 (1.36, 3.11)	12.03 (7.80, 17.42)	14.88 (10.57, 20.76)	588.28 (380.87, 831.55)	960.42 (692.65, 1324.29)
1990	1.38 (0.87, 2.23)	1.96 (1.26, 3.22)	9.26 (5.94, 14.27)	11.69 (7.47, 18.40)	666.54 (361.65, 1044.47)	926.44 (599.13, 1471.36)
**Tropical Latin America**						
2005	2.38 (1.92, 3.02)	2.50 (2.03, 3.13)	14.37 (12.02, 17.65)	14.27 (11.87, 17.21)	893.67 (640.52, 1146.13)	863.62 (659.20, 1086.22)
2021	2.66 (2.28, 3.19)	3.07 (2.61, 3.66)	15.61 (13.69, 17.87)	16.93 (15.04, 19.42)	902.33 (679.25, 1109.90)	1,019.92 (826.78, 1222.27)
1990	2.29 (1.89, 2.82)	2.41 (1.97, 2.96)	11.35 (9.72, 13.47)	10.91 (9.23, 13.03)	793.62 (587.42, 999.56)	734.64 (590.05, 902.43)
**Western Europe**						
2005	1.53 (1.34, 1.78)	1.98 (1.76, 2.26)	13.24 (12.36, 14.44)	16.90 (15.64, 18.26)	2,032.64 (1,592.02, 2343.51)	2,877.67 (2,501.22, 3141.58)
2021	1.32 (1.13, 1.52)	1.69 (1.44, 2.00)	10.88 (9.70, 12.13)	13.93 (12.37, 15.65)	1,692.61 (1,299.09, 1952.32)	2,459.33 (2,107.37, 2754.43)
1990	2.04 (1.80, 2.38)	2.54 (2.21, 2.87)	17.32 (16.07, 18.94)	20.78 (19.21, 22.35)	2290.02 (1829.42, 2627.16)	3038.82 (2670.01, 3333.27)
**Western Sub-Saharan Africa**						
2005	2.05 (1.27, 3.27)	1.74 (1.08, 2.54)	8.27 (5.58, 13.05)	7.38 (4.74, 10.69)	337.37 (213.85, 548.65)	479.42 (283.37, 704.56)
2021	2.39 (1.47, 3.59)	1.81 (1.10, 2.88)	11.83 (7.60, 17.54)	9.13 (5.75, 14.04)	386.31 (231.63, 593.99)	600.87 (363.63, 929.44)
1990	2.55 (1.59, 4.14)	2.06 (1.31, 3.32)	10.53 (6.96, 15.69)	8.86 (5.58, 14.14)	327.08 (202.71, 510.62)	455.89 (286.00, 731.50)

EOCRC – early-onset colorectal cancer, MR – mortality rate, LOCRC – late-onset colorectal cancer, VEOCRC – very early-onset colorectal cancer

*Values are presented as mortality rates per 100,000 persons (95% uncertainty intervals).

Overall, across the globe and the 21 GBD regions in 1990, 2005, and 2021, CRC MRs showed a consistent age gradient, with the highest rates for LOCRC, followed by EOCRC and VEOCRC. All CRC subtypes exhibited distinct sex differences, with substantial geographic variation in MRs across regions.

### Variation in CRC mortality by socioeconomic development levels

In 1990, 2005, and 2021, the high-middle SDI region consistently had the highest MRs across females, males, and all CRC subtypes (**Figure 1**, Panels A and B). The regions with the lowest female VEOCRC MRs varied over time, with the lowest rates observed in the low-middle SDI region in 1990 and the high SDI region in 2021. The lowest EOCRC MRs for both males and females were observed in the low SDI region in 2005. For LOCRC, the lowest MRs were observed among females in the low-middle SDI region and among males in the Low SDI region in 1990.

**Figure 1:**
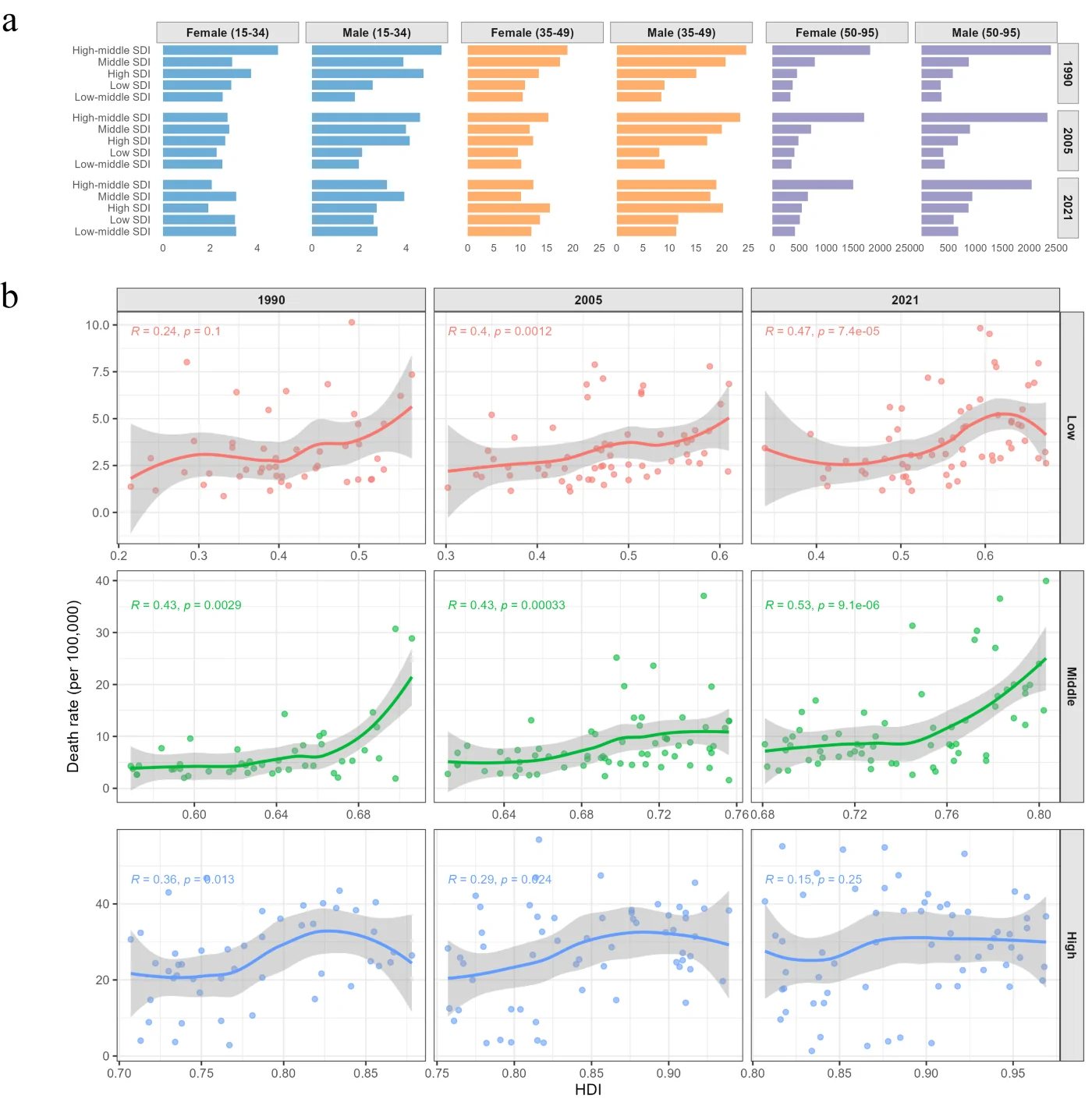
CRC MRs across SDI regions and associations between HDI and overall CRC mortality in 1990, 2005, and 2021. **Panel A.** MRs of VEOCRC, EOCRC, and LOCRC by sex across SDI regions. **Panel B.** Association between HDI and CRC MRs across HDI levels. CRC – colorectal cancer, EOCRC – early-onset colorectal cancer, HDI – Human Development Index, LOCRC – late-onset colorectal cancer, MR – mortality rate, SDI – sociodemographic index, VEOCRC – very early-onset colorectal cancer.

Within the low-HDI group, regional CRC MRs were positively correlated with HDI values in 2005 (*r* = 0.40; *P* = 0.0012) and 2021 (*r* = 0.47; *P* = 7.4 × 10^−5^). In the middle-HDI group, CRC MRs were positively correlated with HDI in 1990 (*r* = 0.43; *P* = 0.0029), 2005 (*r* = 0.40; *P* = 3.3 × 10^−4^), and 2021 (*r* = 0.53; *P* = 9.1 × 10^−6^). In the high-HDI group, CRC MRs were positively correlated with HDI values in 1990 (*r* = 0.36; *P* = 0.013) and 2005 (*r* = 0.29; *P* = 0.024).

We found substantial heterogeneity in CRC mortality across SDI levels, CRC subtypes, sexes, and time points, highlighting disparities in CRC mortality burden by socioeconomic development. Similarly, the association between HDI and CRC mortality varied across HDI strata, with stronger positive correlations observed in low- and middle-HDI regions and weaker associations in high-HDI regions.

### Mortality burden of CRC subtypes in 204 countries and territories

Across the 204 countries and territories, LOCRC consistently had the highest MRs, followed by EOCRC, whereas VEOCRC had the lowest MRs in 1990, 2005, and 2021 (**Figure 2**, Panels A and B). This ranking pattern remained consistent across all three time points. Within each CRC subtype and at each time point, males generally exhibited higher MRs than females. Geographically, CRC MRs were highest in North America, Europe, Oceania, and parts of East Asia, while sub-Saharan Africa had lower MRs.

**Figure 2:**
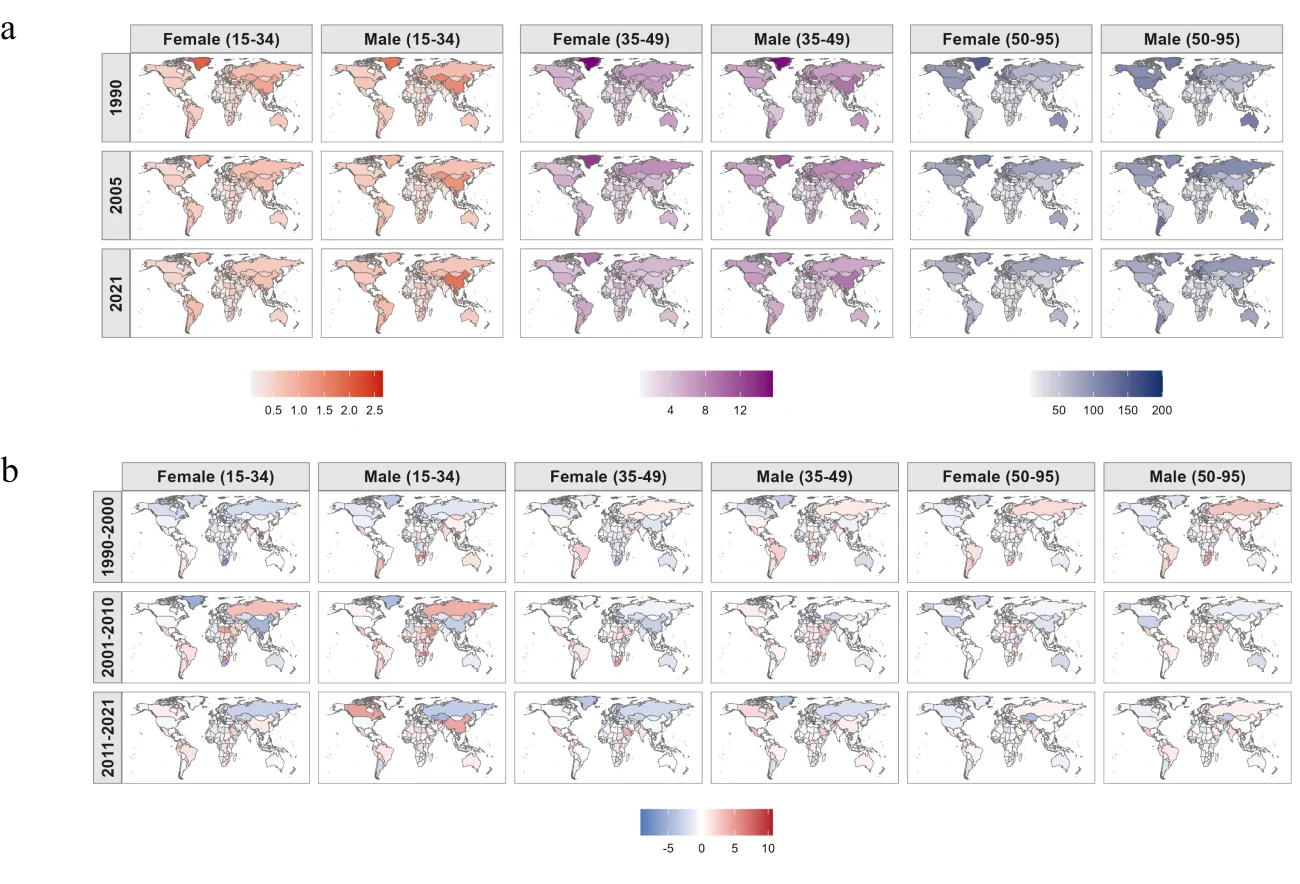
CRC MRs and EAPCs across 204 countries and territories from 1990 to 2021. **Panel A.** MRs of VEOCRC, EOCRC, and LOCRC across 204 countries and territories. **Panel B.** EAPCs of VEOCRC, EOCRC, and LOCRC across 204 countries and territories. CRC – colorectal cancer, EAPC – estimated annual percentage change, EOCRC – early-onset colorectal cancer, HDI – Human Development Index, LOCRC – late-onset colorectal cancer, MR – mortality rate, SDI – sociodemographic index, VEOCRC – very early-onset colorectal cancer.

In most countries, VEOCRC mortality remained stable or declined, although some countries in Asia, Latin America, and Africa showed increasing trends, with positive EAPCs more widely distributed among males. There were pronounced regional disparities in EOCRC, with declining mortality trends predominantly in high-income countries, while increasing trends persisted in some low- and middle-income regions. The greatest spatial heterogeneity was observed for LOCRC, with continued mortality declines in traditional high-burden regions, while increasing trends remained in East Asia, Eastern Europe, Latin America, and some developing regions.

Collectively, CRC mortality exhibited substantial spatial heterogeneity across the 204 countries and territories and showed a strong age gradient. The burden of CRC mortality varied considerably across age groups, with VEOCRC and EOCRC showing more dynamic patterns, whereas LOCRC consistently remained the dominant contributor to the global CRC mortality burden. From 1990 to 2021, CRC mortality trends diverged by region, with overall declines in high-income regions but persistent increases in some developing regions. More pronounced increases were observed among males and older populations.

### Global and SDI-level risk attribution across CRC subtypes

In VEOCRC mortality, the combined PAF for all modifiable risk factors was generally low globally and across all SDI quintiles (**Figure 3**). Overall risk attribution patterns were broadly consistent across SDI levels. Among behavioural risk factors, PAFs attributable to high alcohol consumption were consistently slightly higher in males than in females across all SDI quintiles. Comparing the three time points (*i.e.* 1990, 2005, and 2021) showed no substantial increases in PAFs for any risk factor across sexes or SDI quintiles, and the mortality burden attributable to modifiable risk factors remained relatively stable over time.

**Figure 3:**
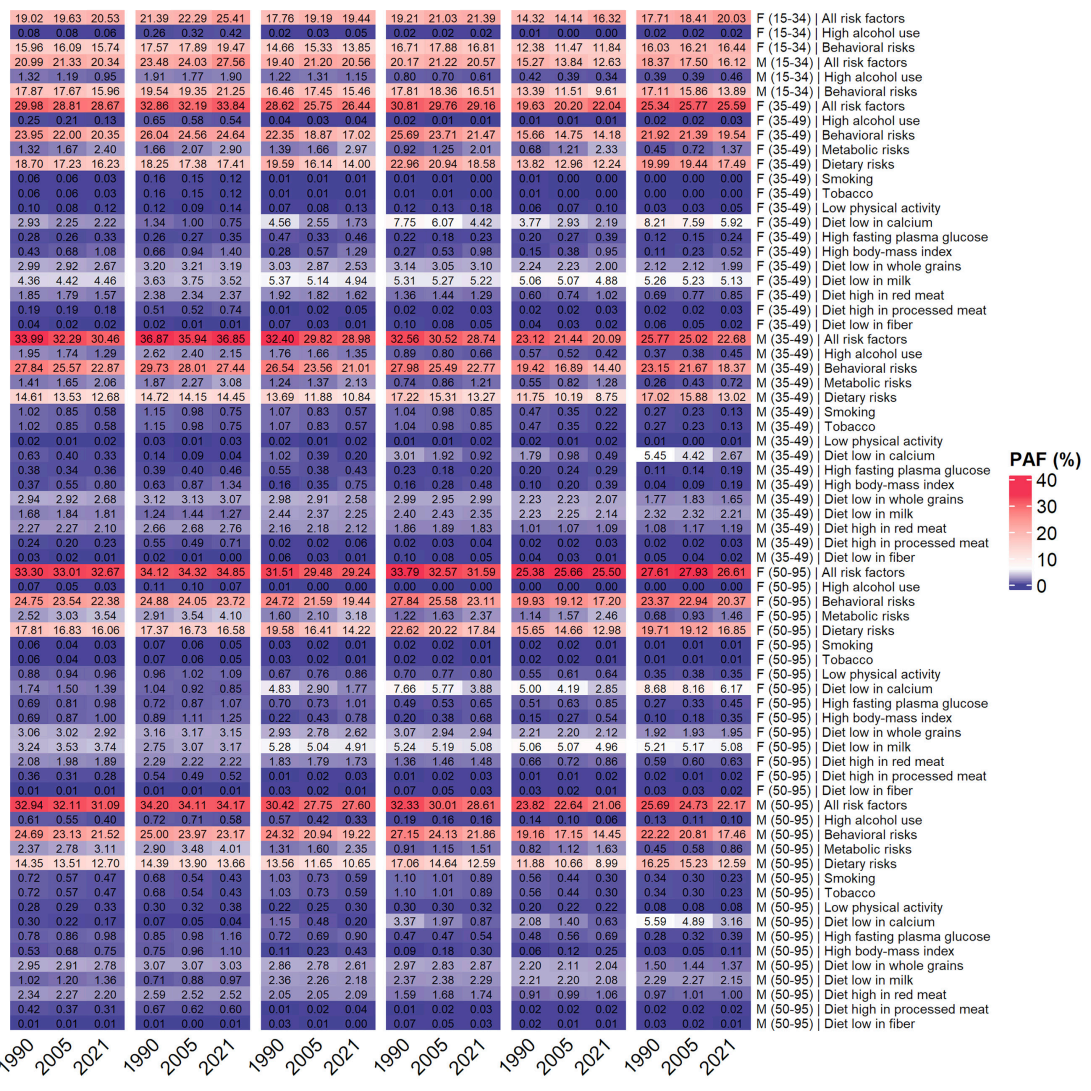
Sex-specific PAFs of risk factors for CRC mortality globally and across five SDI regions. The columns show global, high SDI, high-middle SDI, middle SDI, low-middle SDI, and low SDI PAFs (from left to right). CRC – colorectal cancer, F – female, M – male, PAF – population attributable fraction, SDI – sociodemographic index.

In EOCRC, combined PAFs for all modifiable risk factors were substantially higher than in VEOCRC, and this pattern held globally and across SDI quintiles. High alcohol consumption remained one of the major attributable risk factors, while the contribution of smoking increased markedly and exhibited pronounced sex differences, with higher smoking-related PAFs in males than in females across all SDI quintiles. Meanwhile, metabolic and dietary risk factors, including high fasting plasma glucose, high BMI, low whole grain intake, and high processed meat consumption, showed increasing contributions, whereas low physical activity remained a limited contributor. From 1990 to 2021, PAFs for alcohol consumption, smoking, and metabolic risks increased, indicating a growing contribution of modifiable factors to EOCRC mortality.

In the LOCRC group, the combined PAF of all modifiable risk factors was highest among the three age groups, and this pattern was consistent globally and across all SDI quintiles. High alcohol consumption remained an important attributable risk factor across SDI levels and sexes, while smoking-related PAFs were relatively higher among males, with greater sex disparities observed. Metabolic risks, including high fasting plasma glucose and high BMI, along with dietary and lifestyle factors such as high red meat and processed meat intake, low fibre intake, and low physical activity, showed substantial attributable contributions. Notably, low physical activity contributed significantly more to LOCRC mortality than in younger age groups. At each SDI level and time point, males consistently had higher combined PAFs for all risk factors than females, indicating that modifiable risk factors contributed more to LOCRC mortality among males.

The contribution of modifiable risk factors to CRC mortality increased with older age groups. The combined PAFs were relatively low for VEOCRC mortality, whereas behavioural, metabolic, and dietary risk factors gradually increased their contribution to EOCRC and LOCRC mortality. In addition, males consistently had higher combined PAFs than females across all age groups, indicating that modifiable risk factors contributed more to CRC mortality among males.

### Projected global CRC mortality trends by subtype

During the projection period, ASMRs for all CRC subtypes were projected to decline. LOCRC consistently had the highest ASMRs, followed by EOCRC, while VEOCRC had the lowest rates; this gradient remained stable throughout the projection period. By 2050, rates for females with VEOCRC (ASMR = 0.31; 95% UI = −0.09, 0.71), EOCRC (ASMR = 2.71; 95% UI = −0.33, 5.75), and LOCRC (ASMR = 31.40; 95% UI = 6.51, 56.28) were projected to decrease. Similarly, rates for males with VEOCRC (ASMR = 0.41; 95% UI = −0.18, 0.98), EOCRC (ASMR = 4.91; 95% UI = −1.82, 11.63), and LOCRC (ASMR = 57.47; 95% UI = 13.06, 101.90) were also projected to decrease. Overall, males consistently exhibited higher ASMRs than females across all CRC subtypes, whereas projected declines were more pronounced among females, particularly for LOCRC. Uncertainty increased with the projection horizon across all CRC subtypes, and the 95% UIs were generally wider for males than for females.

The BAPC projections indicate that the global ASMR of CRC is expected to decline or remain relatively stable by 2050, with age-related patterns and sex differences remaining consistent over time (Figure S1 in the **Online Supplementary Document**). The LOCRC consistently showed the highest MRs, followed by EOCRC and VEOCRC, and males were projected to have higher MRs than females across all CRC subtypes.

## DISCUSSION

Using GBD 2021 data, we systematically assessed the mortality burden of VEOCRC, EOCRC, and LOCRC worldwide from 1990 to 2021 from a life-course perspective, and further evaluated the contributions of risk factors and future trends. We found substantial age-, sex-, and geography-related heterogeneity in CRC mortality burden. LOCRC consistently accounted for the largest proportion of global CRC mortality, whereas VEOCRC and EOCRC showed more dynamic patterns. Marked disparities emerged across regions with different socioeconomic development levels, reflecting a complex association between CRC mortality burden and development status. Risk attribution analyses showed that the contribution of modifiable risk factors to CRC mortality increased with advancing age, with males being more strongly affected by these risk factors. The BAPC projections indicated that global CRC mortality is expected to continue declining through 2050; however, age gradients and sex disparities are likely to persist, highlighting the need for tailored CRC prevention and control strategies across different age groups.

Existing studies have primarily focused on EOCRC (<50 years), which can be further classified into VEOCRC (15–34 years) and EOCRC (35–49 years). Although mechanistic studies specifically investigating these subgroups remain limited, their risk factors and underlying biological mechanisms show substantial overlap. The development of VEOCRC and EOCRC is unlikely to be driven by a single factor but rather results from complex interactions among genetic susceptibility, early-life exposures, metabolic abnormalities, dietary and lifestyle changes, gut microbiota dysbiosis, and molecular alterations [26]. The increased mortality risk among patients with EOCRC (<50 years) may be attributable to delayed diagnosis resulting from the lack of routine screening, as well as the potentially more aggressive biological characteristics of these tumours. Because younger individuals are generally not included in population-based CRC screening programmes and may be less likely to recognise symptoms such as rectal bleeding, abdominal pain, and changes in bowel habits, diagnosis is often delayed until the disease has progressed to an advanced stage [27]. In addition, EOCRC (<50 years) more commonly presents at advanced stages and has aggressive histopathological features, including mucinous adenocarcinoma, signet-ring cell carcinoma, and, in a subset of patients, microsatellite instability-high/deficient mismatch repair tumours. These characteristics may contribute to poorer prognosis and an increased risk of mortality [28]. Furthermore, genetic susceptibility plays an important role in young patients with CRC. Approximately 16% of young patients with CRC carry pathogenic germline variants, primarily involving hereditary cancer syndromes such as Lynch syndrome, *APC*-associated familial adenomatous polyposis, and *MUTYH*-associated polyposis, highlighting the important contribution of inherited predisposition to the development of EOCRC (<50 years) [29]. Obesity, unhealthy dietary patterns, physical inactivity, and other modifiable risk factors also contribute to the development of EOCRC (<50 years) [30].

Previous studies have focused primarily on LOCRC, largely because it has historically accounted for most of the global CRC burden. The substantial mortality burden of LOCRC is driven by population ageing and the cumulative effects of lifelong exposure to established risk factors and age-related biological changes [31]. With advancing age, accumulated genetic damage, epigenetic changes, and chronic inflammation may contribute to CRC development and progression, thereby increasing mortality risk [4]. In addition, older patients often have a greater burden of comorbidities, reduced physiological reserve, and lower tolerance to intensive treatments, which may further limit therapeutic effectiveness and increase the risk of mortality [32].

The higher MRs of EOCRC and LOCRC among males may be partly explained by greater exposure to modifiable risk factors. Compared with females, males generally have higher exposure to smoking, high alcohol consumption, high intake of red and processed meat, obesity, and insufficient physical activity, which may contribute to CRC development and progression through mechanisms involving chronic inflammation, metabolic dysregulation, and tumour-promoting pathways [33]. In addition, sex-specific differences in lifestyle behaviours and endogenous hormonal profiles may further contribute to the observed sex disparities in CRC incidence and mortality [34].

A study based on data from International Agency for Research on Cancer registries and World Health Organization mortality databases showed that CRC mortality patterns and temporal trends were closely associated with human development levels, with the observed gradual transitions potentially reflecting the increasing adoption of Westernised lifestyles accompanying socioeconomic development [35]. The decline in CRC mortality in high-income countries is likely attributable to higher screening coverage and more comprehensive healthcare systems, whereas low- and middle-income countries have shown less pronounced declines, with some regions continuing to experience increasing mortality, potentially related to limited screening coverage, constrained healthcare resources, and reduced access to precision medicine [36]. Differences in CRC mortality burden across regions with varying socioeconomic development levels may reflect disparities in risk factor exposure and healthcare resources. Regions with higher socioeconomic development generally have greater health literacy, stronger preventive capacity, and more comprehensive healthcare systems, whereas less developed regions often face greater exposure to risk factors and limited healthcare resources.

Future declines in CRC mortality may reflect improved screening coverage, earlier diagnosis, and advances in treatment. However, age-related genetic and epigenetic alterations, chronic inflammation, and cumulative exposure to established risk factors throughout the life course may continue to contribute to the substantially higher mortality burden of LOCRC than EOCRC and VEOCRC. The higher MRs among males may reflect greater exposure to modifiable risk factors and sex-related hormonal differences.

### Limitations

Several limitations should be acknowledged. First, GBD data are derived from modelling estimates, and differences in data quality, completeness of mortality registration, and modelling assumptions across regions may influence the results. Second, PAF analyses based on the GBD comparative risk assessment framework reflect population-level attributable contributions rather than individual-level causal effects. Moreover, this framework includes only a subset of established risk factors and does not account for potential determinants such as genetic susceptibility, family history, gut microbiota, and other environmental exposures. In addition, interactions among risk factors and uncertainty in exposure–outcome relationships may affect estimates of attributable risk. Third, although stratifying CRC into VEOCRC, EOCRC, and LOCRC provides a practical framework for evaluating age-related heterogeneity from a life-course perspective, these predefined age categories may not fully capture the continuous biological processes underlying CRC development and progression. Furthermore, the relatively small number of deaths among younger age groups and limited data availability in some regions may increase uncertainty in the estimates. Finally, BAPC projections rely primarily on historical age–period–cohort effects and do not account for potential future changes in risk factor exposure, screening strategies, therapeutic advances, or healthcare accessibility. The lack of independent external validation also limits long-term projection analyses; therefore, interpret projected trends with caution. Therefore, the projected trends should be interpreted with caution.

## CONCLUSIONS

We found substantial age-, sex-, and regional heterogeneity in the global CRC mortality burden during 1990–2050. Although global CRC mortality is projected to continue declining, LOCRC will remain the predominant contributor to the overall mortality burden, while the changing patterns of VEOCRC and EOCRC highlight the need for increased attention to CRC prevention and control among younger populations. The contributions of modifiable risk factors to CRC mortality varied across age groups, with higher attributable fractions observed among males. Therefore, future CRC control should integrate continued improvements in screening and treatment with age- and sex-specific interventions that target modifiable risk factors and promote tailored prevention strategies.

## Additional material


Online Supplementary Document

## Data Availability

**Data availability:** The GBD data used in the analyses are available at https://ghdx.healthdata.org/gbd-results-tool. Additional data supporting this article can be obtained upon request to the corresponding author.
