# Supplementary material for: Global burden of colorectal cancer mortality by age and sex, 1990–2050: a population-based analysis
Source: J Glob Health. 2026 Nov 6;16:04383. doi: 10.7189/jogh.16.04383 (PMC13741170; doi:10.7189/jogh.16.04383)

Supplement to: Luo T, Chen W, Cao Y, Li P, Feng J. Global burden of colorectal cancer **mortality by age and sex, 1990–2050: a population-based analysis. J Glob** Health.2026;16:04383.

Table S1. Adherence to JoGH's GRABDROP guidelines items

**JoGH guideline items**
1. Please list all papers published by each co-author in previous 3 years that were based on secondary analysis of a big data repository
Feng J, Pan C, Gou Y, et al. EAT-Lancet Diet Modifies the Risk of Rheumatoid Arthritis Through Metabolomic Signature. *Arthritis Rheumatol*. 2026;78(1):68-79.
2. Please explain the key elements of your study design and the use of the available datasets that make your study an original scientific contribution
This study used data from the Global Burden of Disease Study 2021 to systematically evaluate colorectal cancer mortality burden by age at onset (very early-onset CRC, early-onset CRC, and late-onset CRC), sex, and geographic location from 1990 to 2021, with projections to 2050. Unlike previous studies focusing on overall CRC burden, this study adopted a life-course perspective, integrated risk factor attribution analyses, and assessed future mortality trajectories using Bayesian age–period–cohort models.
3. Please list all publications that addressed similar research questions in the same dataset and indicate where you cited them in your paper
1. Wang Z, Yao W, Wu W, et al. Global incidence trends of early-onset colorectal cancer and related exposures in early-life: an ecological analysis based on the GBD 2019. Front Public Health 2024; 12: 1367818.<br>2. Li X, Xiao X, Wu Z, Li A, Wang W, Lin R. Global, regional, and national burden of early-onset colorectal cancer and projection to 2050: An analysis based on the Global Burden of Disease Study 2021. Public Health 2025; 238: 245-53.<br>3. Zhang J, Ou D, Xie A, Chen D, Li X. Global burden and cross-country health inequalities of early-onset colorectal cancer and its risk factors from 1990 to 2021 and its projection until 2036. BMC Public Health 2024; 24(1): 3124.<br>4. Liang Y, Zhang N, Wang M, et al. Distributions and Trends of the Global Burden of Colorectal Cancer Attributable to Dietary Risk Factors over the Past 30 Years. Nutrients 2023; 16(1).<br>5. Jin X, Bai B, Pan Y, et al. Distinct clinical features and epidemiological trends of very early and middle-aged early onset colorectal cancer based on SEER 17 and GBD 2021 data over the past 15 years. BMC Cancer 2025; 25(1): 1923.<br>Previous GBD-based studies on colorectal cancer have mainly assessed overall incidence, mortality, DALYs, and temporal trends across countries and regions. These studies were cited in the Introduction section. Unlike previous analyses, our study adopts a life-course perspective by stratifying CRC according to age at onset (VEOCRC, EOCRC, and LOCRC) and integrates sex-specific analyses, risk factor attribution, and BAPC projections to provide a more comprehensive assessment of global CRC mortality patterns.
4. Please explain how you addressed multiple testing through an appropriately rigorous statistical

threshold and indicate this in the methods section
As this study primarily described population-level trends and burden estimates rather than testing multiple hypotheses, adjustment for multiple comparisons was not applicable.
5. Please declare to what extent have AI chatbots been used in developing your paper and to which parts of the paper did they contribute
No AI chatbots were used in the development, analysis, interpretation, or writing of this manuscript.

Figure S1. Observed and projected global ASMRs of three CRC subtypes by sex using the BAPC model. Shaded areas represent the 95% UIs.

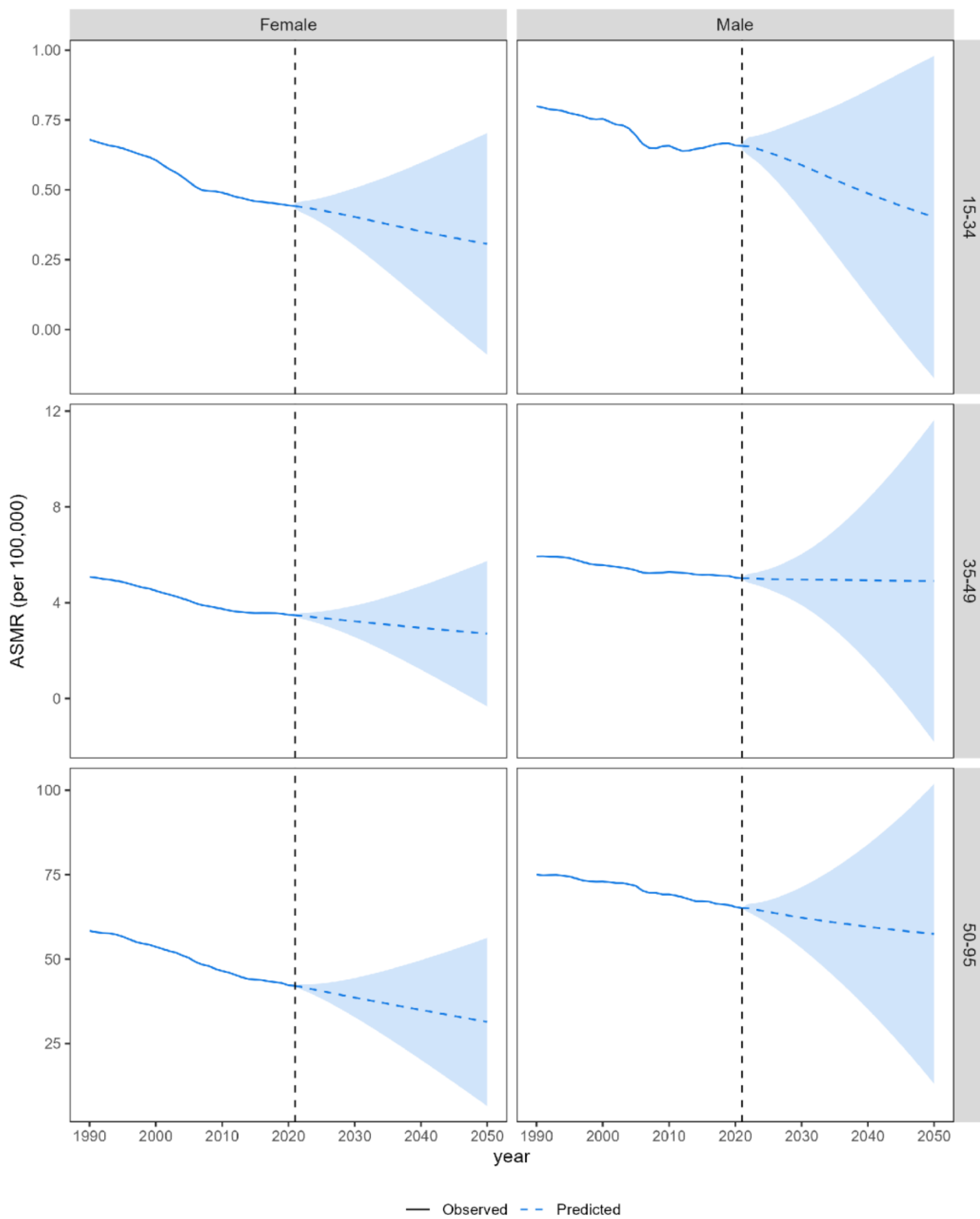

Supplement: Supplementary file 1 [file jogh-16-04383-s001.pdf]
